# Changes in maternal age and prevalence of congenital anomalies during the enactment of China's universal two-child policy (2013–2017) in Zhejiang Province, China: An observational study

**DOI:** 10.1371/journal.pmed.1003047

**Published:** 2020-02-24

**Authors:** Xiaohui Zhang, Lijin Chen, Xuemiao Wang, Xiaoyan Wang, Menghan Jia, Saili Ni, Wei He, Shankuan Zhu

**Affiliations:** 1 Chronic Disease Research Institute, School of Public Health, and Women’s Hospital, Zhejiang University School of Medicine, Hangzhou, Zhejiang, China; 2 Department of Nutrition and Food Hygiene, School of Public Health, Zhejiang University, Hangzhou, Zhejiang, China; 3 Department of Women’s Health, Women’s Hospital, Zhejiang University School of Medicine, Hangzhou, Zhejiang, China; 4 Department of Medical Epidemiology and Biostatistics, Karolinska Institutet, Stockholm, Sweden; University of Manchester, UNITED KINGDOM

## Abstract

**Background:**

China implemented a partial two-child policy (2013) followed by a universal two-child policy (2015), replacing the former one-child policy mandated by the government. The changes affect many aspects of China’s population as well as maternal and infant health, but their potential impact on birth defects (BDs) remains unknown. In this study, we investigated the associations of these policy changes with BDs in Zhejiang Province, China.

**Methods and findings:**

We used data from the BD surveillance system in Zhejiang Province, China, which covers 90 hospitals in 30 urban districts and rural counties, capturing one-third of the total births in this province. To fully consider the time interval between conception and delivery, we defined the one-child policy period as data from 2013 (births from October 2012 to September 2013), the partial two-child policy period as data from 2015 (births from October 2014 to September 2015), and the universal two-child policy period as data from 2017 (births from October 2016 to September 2017). Data from 2009 and 2011 were also used to show the changes in the proportion of births to women with advanced maternal age (35 years and older) prior to the policy changes. Main outcome measures were changes in the proportion of mothers with advanced maternal age, prevalence of BDs, rankings of BD subtypes by prevalence, prenatal diagnosis rate, and live birth rate of BDs over time. A total of 1,260,684 births (including live births, early fetal losses, stillbirths, and early neonatal deaths) were included in the analyses. Of these, 644,973 (51.16%) births were to women from urban areas, and 615,711 (48.84%) births were to women from rural areas. In total, 135,543 (10.75%) births were to women with advanced maternal age. The proportion increased by 85.68%, from 8.52% in 2013 to 15.82% in 2017. However, it had remained stable prior to policy changes. Overall, 23,095 BDs were identified over the policy changes (2013–2017). The prevalence of BDs during 2013, 2015, and 2017 was 245.95, 264.86, and 304.36 per 10,000 births, respectively. Trisomy 21 and other chromosomal defects increased in both risk and ranking from 2013 to 2017 (crude odds ratio [95% confidence interval] 2.13 [1.75–2.60], from ranking 10th to 5th, and 3.63 [2.84–4.69], from ranking 16th to 6th, respectively). The prenatal diagnosis rate increased by 3.63 (2.2–5.1) percentage points (*P* < 0.001), from 31.10% to 34.72%, and identification of BDs occurred 1.88 (1.81–1.95) weeks earlier (*P* < 0.001). The live birth rate for infants with BDs born before 28 gestational weeks increased from 1.29% to 11.45%. The major limitations of this observational study include an inability to establish causality and the possible existence of unknown confounding factors, some of which could contribute to BDs.

**Conclusions:**

In this study, we observed significant increases in maternal age and the prevalence of total and age-related anomalies following China’s new two-child policy. Increases in live birth rate for infants with BDs born before 28 gestational weeks suggest that healthcare for very preterm births with BDs may be warranted in the future, as well as updating the definition of perinatal period.

## Abstract 2

In an observational study, Xiaohui Zhang and colleagues investigate the changes in maternal age and prevalence of congenital anomalies during the enactment of China's universal two-child policy (2013-2017) in Zhejiang Province, China.

## Author summary

**Why was this study done?:**

- China's birth policy has changed dramatically in recent years, from a one-child policy to a partial two-child policy to the current universal two-child policy. These changes affect many aspects of the Chinese population as well as maternal health.
- Birth defects (BDs) or congenital anomalies are structural or functional anomalies occurring in a fetus or newborn and are the leading causes of infant death globally. There are considerable debates on the effects of China's birth policy changes. However, the associations of these changes with BDs are unclear.

**What did the researchers do and find?:**

- We used data from the BD surveillance system of Zhejiang Province, including 1.26 million births, to investigate the associations of birth policy changes with BDs.
- We found that the proportion of births to women with advanced maternal age (35 years and over) increased by 85.68% with policy changes, from 8.52% in 2013 to 15.82% in 2017. We also found obvious increases in the prevalence of total and age-related anomalies.
- The prenatal diagnosis rate also increased over time. Furthermore, the live birth rate for infants with BDs born before 28 gestational weeks increased sharply.

**What do these findings mean?:**

- Our findings suggest that, with relaxation of China’s one-child policy, greater attention is needed for women with advanced maternal age and infants with BDs born before 28 gestational weeks.
- The major limitations of this observational study include an inability to establish causality and the possible existence of unknown confounding factors, some of which could contribute to BDs.

## Introduction

Because China had a resident population of more than 962 million people in 1979 [1], the Chinese government initiated a one-child policy in that year based on fears of overpopulation and related economic, social, and environmental challenges [2]. However, rapid socioeconomic development, a decline in the total fertility rate, pressures owing to an aging population, sex-ratio imbalance, and workforce shortages over recent decades have resulted in increased public awareness of these issues [3]. In November 2013, the Chinese government launched a partial two-child policy, which encouraged couples to have a second child if either parent was a singleton. Following this, a universal two-child policy was officially declared in October 2015, meaning that all families in China are entitled to have two children [4]. By 2016, hospital surveillance data indicated that the number of live births had soared to 17.86 million per year, reflecting a 7.9% increase over 2015 [5].

Some questions remain about the impacts of the birth policy changes on maternal and infant health. To date, the impact of birth policy changes on women’s reproductive decisions, delivery mode, pregnancy complications, pregnancy age, and birth outcomes has been reported [6–11]. An association between certain maternal characteristics and birth defects (BDs) has also been reported, such as maternal age, maternal parity, use of assisted reproductive technology, and diabetes mellitus [12–15]. However, few studies have conducted a comprehensive investigation of the epidemiology of BDs with respect to the birth policy changes in China. BDs or congenital anomalies are defined as structural or functional anomalies occurring in a fetus or newborn and are the leading causes of infant death globally [16–20].

Zhejiang Province is located in southeastern China and is at the forefront of the country’s socioeconomic development, boasting the country’s leading maternal and child health system. In 2015, Zhejiang Province had a resident population of 55.39 million and was ranked fourth in the country for its gross regional product (GRP) (USD 688.56 billion). Moreover, the maternal mortality rate in this province was the lowest in China [1,21]. A BD surveillance system has been in operation in the province since 1990. In this study, we explored and compared BDs in all births before and after the birth policy changes using data from the BD surveillance system of Zhejiang Province, China. We aimed to gain a better understanding of BDs among infants born at all gestational weeks and whether these might be associated with the birth policy changes.

## Methods

### Study design and population

This was an observational study based on monitoring data from the provincial BD surveillance system in Zhejiang Province, China. To investigate the associations of BDs and policy changes, we planned to conduct this study after obtaining data for 2017. There was no prespecified analysis plan. To fully consider the time interval between conception and delivery, we defined the period of the one-child policy (introduced in 1979) as the data from 2013 (births from October 2012 to September 2013), that of the partial two-child policy (introduced in November 2013) as the data from 2015 (births from October 2014 to September 2015), and the period of the universal two-child policy (introduced in October 2015) period as the data from 2017 (births from October 2016 to September 2017). The system records all births, including live birth, early fetal loss (embryonic or fetal death before 28 gestational weeks), stillbirths (fetal death at 28 gestational weeks or later), and early neonatal deaths (infant death within 7 days after birth). In this study, a total of 1,260,684 births were included. Of these, 644,973 (51.16%) births were to women from urban areas, and 615,711 (48.84%) births were to women from rural areas. In total, 135,543 (10.75%) births were to women aged 35 years and over. Among the total births, 844,503 births were recorded in the surveillance system for the years 2013, 2015, and 2017, representing the periods of birth policy changes in China. The remaining data comprised that from 2009 and 2011, representing periods prior to birth policy changes, to better understand the influence of birth policy changes on maternal age. This study was approved by the Ethics Committee of the Women's Hospital, Zhejiang University School of Medicine (number: 2018KY036).

### Data collection

We used data from a hospital-based BD surveillance system in Zhejiang Province. The system covers 90 hospitals from 30 of 89 urban districts and rural counties in this province. The sampling strategy for hospital surveillance was as follows: There are 11 cities in Zhejiang Province. In each city, two to four districts and counties were recruited according to population size and rural and urban distribution. In each selected district or county, hospitals were recruited according to number of births. Annual births captured in this system included one-third of total births in Zhejiang Province. For births with BDs, individual information was collected. However, for births without BDs, no information at the individual level was recorded; only the distribution of birth characteristics was obtained. Mothers of the infants included in this study received routine prenatal care visits at least five to 10 times throughout their pregnancy, according to the prenatal care regulation released by China's Ministry of Health [22]. Individual information relating to births with BDs was collected using a structured form, which included maternal characteristics, antenatal screening, BD diagnosis, birth information, and birth outcomes. Pediatricians and obstetricians with standardized training in BD diagnosis and data collection reviewed the clinical records and extracted data from the maternal health information using the form. Quality control with respect to data completeness, coverage, and validity was performed monthly by staff of the surveillance hospitals, twice a year in local maternal and child health hospitals, and annually at the Women’s Hospital of Zhejiang University School of Medicine.

### Criteria of BD diagnosis and variables definition

BDs were diagnosed by obstetricians and pediatricians using ultrasound, genetic, pathology, and laboratory testing. The diagnoses covered 25 BD subtypes based on the International Statistical Classification of Diseases and Related Health Problems 10th Revision (Q00-Q99) [23], including congenital heart defects (CHDs), congenital malformation of the urinary system, cleft lip with cleft palate, cleft lip without cleft palate, cleft palate without cleft lip, polydactyly, syndactyly, congenital hydrocephalus, congenital talipes equinovarus, congenital microtia, other malformation of the external ear as a whole, trisomy 21 syndrome, other chromosomal abnormalities as a whole, hypospadias, anencephaly, spina bifida, encephalocele, omphalocele, limb reduction defects, congenital atresia of the rectum and anus, congenital diaphragmatic hernia, gastroschisis, congenital esophageal atresia, conjoined twins, and exstrophy of the urinary bladder.

We applied commonly used definitions for maternal characteristics [11,24]. Nulliparous was defined as women who had not previously given birth at or after 28 completed weeks of gestation. Advanced maternal age was defined as women aged 35 years or older at delivery. Early fetal loss was defined as embryonic or fetal deaths before 28 gestational weeks. Stillbirth was defined as fetal deaths at 28 gestational weeks or later. Early neonatal deaths were defined as infant death within 7 days after birth. Multiple congenital anomalies were defined as two or more isolated anomalies occurring in a fetus or newborn.

### Statistical analysis

Continuous variables are presented as means and standard deviation (SD), and categorical variables are presented as number (*N*) and percentage. The prevalence of BDs is presented as the number of BDs per 10,000 births. Changes in the characteristics of total births and births with BDs were tested using analysis of variance (ANOVA) for continuous variables and chi-square tests for categorical variables. Cramér’s V and *η*2 (eta square) were calculated in chi-squared tests and ANOVA, respectively, to estimate effect sizes. Bonferroni correction was performed in multiple comparisons among the time periods. Trend analysis was used to track characteristics over the time frame of policy changes. Line plots and histograms were drawn to show changes in the distribution of women with advanced maternal age over time. In addition to 2013, 2015, and 2017, corresponding surveillance data from 2009 and 2011 were also used in the line plots and histograms to represent periods prior to policy changes in investigating the changes in advanced maternal age. In age-stratified analyses, crude odds ratios (ORs) and 95% confidence intervals (CIs) were calculated to examine the association of policy changes with risk of BDs in each maternal age group, with the one-child policy period being defined as the reference group. BDs with multiple anomalies were divided into subtypes of isolated malformations to calculate the prevalence and live birth rate and ranked in descending order by prevalence. Crude ORs and 95% CIs were also calculated to examine the association of policy changes with the risk of BD subtypes in total births, with the one-child policy period being defined as the reference group. Analysis of BDs was conducted among total BDs, as well as births with BDs occurring before and after 28 gestational weeks, separately, according to the definition of the perinatal period in China [25]. All statistical analyses were performed using Stata version 13 (StataCorp, College Station, TX, United States). *P* values < 0.05 (two-sided) were considered statistically significant and are otherwise indicated.

## Results

### Characteristics of total births and changes in the prevalence of BDs

**Fig 1:**
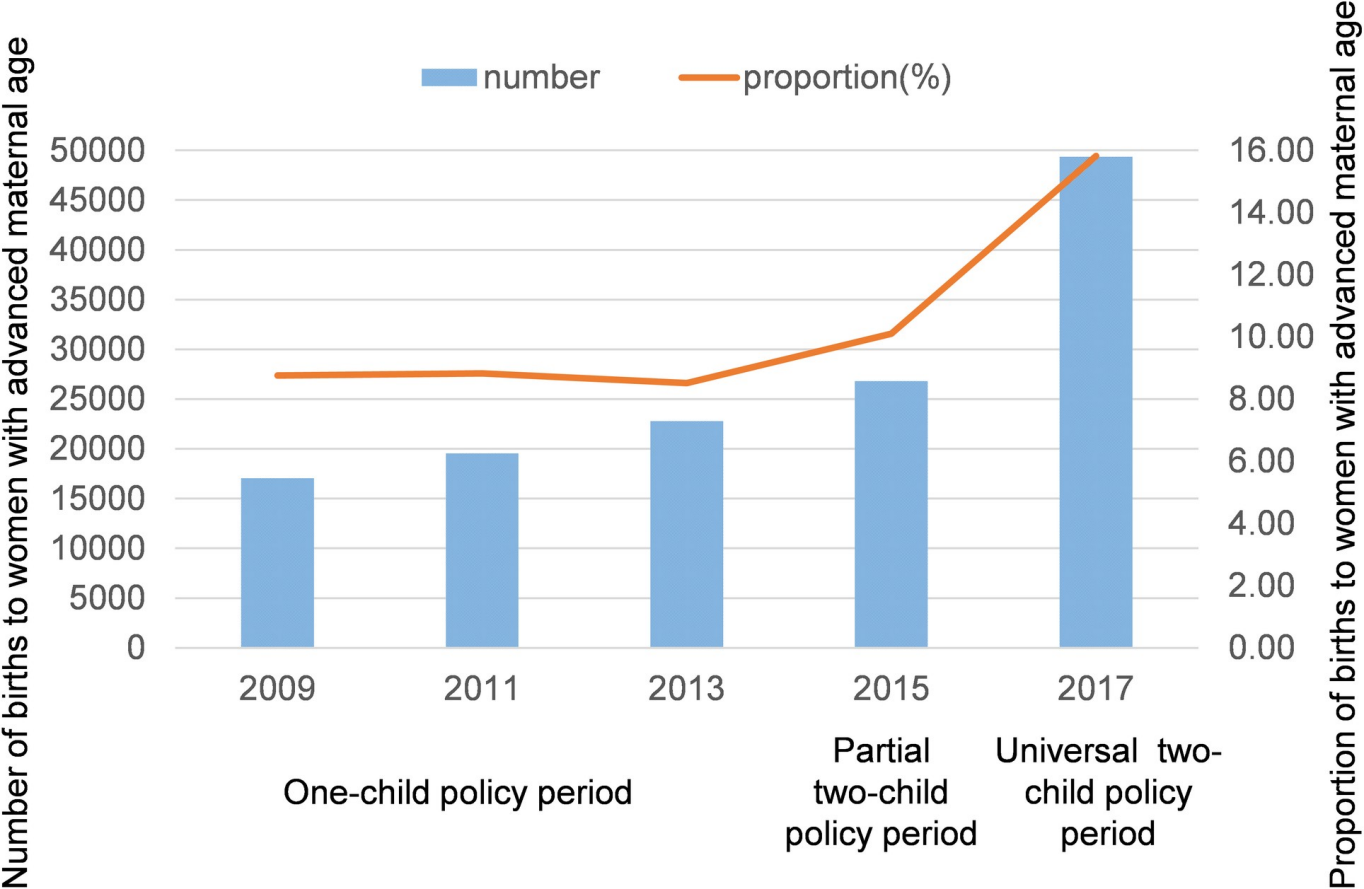
Changes in the number and percentage of births to women with advanced maternal age (≥35 years) before and after policy changes. Bars indicate the number of births to women with advanced maternal age; the line indicates the percentage. Chi-squared tests with Bonferroni correction were performed in multiple comparisons over years. Significant differences in all pairwise comparisons from 2013 to 2017 were observed; no significant increase in all pairwise comparisons from 2009 to 2013 was found (*P* < 0.0125, adjusted using Bonferroni correction, 0.05/4 tests).

A total of 1,260,684 births were included: 194,712 births in 2009, 221,469 births in 2011, 267,126 births in 2013, 265,277 births in 2015, and 312,100 births in 2017. Changes in birth characteristics over the period of policy changes (in 2013, 2015, and 2017) are shown in Table 1. An increase of 44,974 births occurred over this period. We also observed significant changes in the distributions of maternal age, maternal region, infant sex, and number of embryos (all *P* < 0.001). The proportion of women who lived in urban areas and the proportion of female infants increased. The proportion of births to women aged 30–34 and ≥35 years increased, whereas the proportion of births to women aged 20–24 and <20 years decreased from 2013 to 2017. The proportion of births to women aged ≥35 years increased by 85.68% with policy changes, from 8.52% in 2013 to 15.82% in 2017; however, no significant increase in the corresponding data was found from 2009 to 2013 prior to changes in the birth policy (Fig 1). The number of BDs increased from 6,570 during the one-child policy period to 9,499 during the universal two-child policy period, whereas the prevalence of BDs increased from 245.95 per 10,000 births to 304.36 per 10,000 births (OR = 1.24, 95% CI 1.21–1.29, *P* < 0.001) (Table 1). Among BDs, the proportion of multiple anomalies increased from 5.77% during the one-child policy period to 10.36% during the universal two-child policy period (OR = 1.89, 95% CI 1.67–2.14, *P* < 0.001). Among all BDs, the proportion of BDs among infants born before 28 gestational weeks increased from 16.59% during the one-child policy period to 23.31% during the universal two-child policy period (OR = 1.53, 95% CI 1.41–1.66, *P* < 0.001).

**Table 1:** Changes in birth characteristics over the period of policy changes (in 2013, 2015, and 2017).

Variables	One-child policy period (2013)		Partial two-child policy period (2015)		Universal two-child policy period (2017)		Cramér’s V	*P* value
	*N*	Percent	*N*	Percent	*N*	Percent		
**Total births**	267,126	100.00	265,277	100.00	312,100	100.00	**-**	**-**
** Maternal age**							**0.105**	**<0.001**
<20	8,623	3.23	7,841	2.96	5,849	1.87		
20–24	64,392	24.20	51,051	19.34	46,192	14.91		
25–29	116,384	43.75	117,648	44.56	125,566	40.52		
30–34	54,962	20.58	61,935	23.35	85,124	27.27		
≥35	22,765	8.52	26,802	10.10	49,369	15.82		
**Maternal region**							**0.032**	**<0.001**
Urban	136,193	50.98	136,049	51.29	169,789	54.40		
Rural	130,933	49.02	129,228	48.71	142,311	45.60		
Infant sex*							**0.006**	**<0.001**
Male	141,430	52.95	140,268	52.88	163,037	52.24		
Female	125,447	46.96	124,772	47.03	148,693	47.64		
Unknown	194	0.07	208	0.08	331	0.11		
No information	55	0.02	29	0.01	39	0.01		
** Number of embryos**							**0.005**	**<0.001**
Single birth	263,230	98.54	261,028	98.40	307,129	98.41		
Multiple births	3,896	1.46	4,249	1.60	4,971	1.59		
**Total BDs**	6,570	245.95^#^	7,026	264.86^#^	9,499	304.36^#^	**0.015**	**<0.001 (trend)**
** Total**	6,570	100.00	7,026	100.00	9,499	100.00		
** Gestational age**							**0.076**	**<0.001 (trend)**
Born before 28 weeks	1,090	16.59	1,248	17.76	2,214	23.31		
Born at 28 weeks or later	5,480	83.41	5,778	82.24	7,285	76.69		
** Type**							**0.069**	**<0.001 (trend)**
Multiple anomalies	379	5.77	558	7.94	984	10.36		
Isolated anomalies	6,191	94.23	6,468	92.06	8,515	89.64		

*P* values and Cramér’s V were derived from chi-squared tests. *P*_trend_ was derived from trend analyses.

*Unknown indicates that infant sex could not be identified; no information indicates missing data on infant sex.

^#^ Prevalence of total BDs presented as number of total BDs per 10,000 births.

Abbreviation: BD, birth defect

After stratification by maternal age, the higher risk of BDs over the policy change time frame remained significant (Table 2). During the universal two-child policy period, the risk of BDs among births to women in the age groups 20–24, 25–29, 30–34, and ≥35 years was significantly increased compared with during the one-child policy period (20–24 years: OR = 1.38, 95% CI 1.28–1.49, *P* < 0.001; 25–29 years: OR = 1.22, 95% CI 1.16–1.28, *P* < 0.001; 30–34 years: OR = 1.11, 95% CI 1.04–1.18, *P* = 0.002; and ≥35 years: OR = 1.16, 95% CI 1.06–1.27, *P* < 0.001). In the partial two-child policy period, the prevalence of BDs among births to women aged 20–24 years (OR = 1.15, 95% CI 1.07–1.25, *P* < 0.001) and ≥35 years (OR = 1.13, 95% CI 1.02–1.24, *P* = 0.015) was also significantly higher than that during the one-child policy period.

**Table 2:** Crude ORs (95% CI) for the association between birth policy changes and BDs, stratified by maternal age groups (in 2013, 2015, and 2017).

Age groups	One-child policy period (2013)	*P* value	Partial two-child policy period (2015)	*P* value	Universal two-child policy period (2017)	*P* value
Total	1	**-**	**1.08 (1.04**–**1.12)**	**<0.001**	**1.24 (1.21**–**1.29)**	**<0.001**
<20	1	**-**	1.20 (0.99–1.46)	0.060	1.11 (0.90–1.38)	0.319
20–24	1	**-**	**1.15 (1.07**–**1.25)**	**<0.001**	**1.38 (1.28**–**1.49)**	**<0.001**
25–29	1	**-**	1.05 (1.00–1.11)	0.058	**1.22 (1.16**–**1.28)**	**<0.001**
30–34	1	**-**	0.98 (0.91–1.05)	0.546	**1.11 (1.04**–**1.18)**	**0.002**
≥35	1	**-**	**1.13 (1.02**–**1.24)**	**0.015**	**1.16 (1.06**–**1.27)**	**<0.001**

ORs and 95% CIs were calculated from exposures (policy changes) and cases (BDs). *P* values were derived from chi-squared tests.

Abbreviations: BD, birth defect; CI, confidence interval; OR, odds ratio

### Changes in prevalence and ranking of BD subtypes

**Fig 2:**
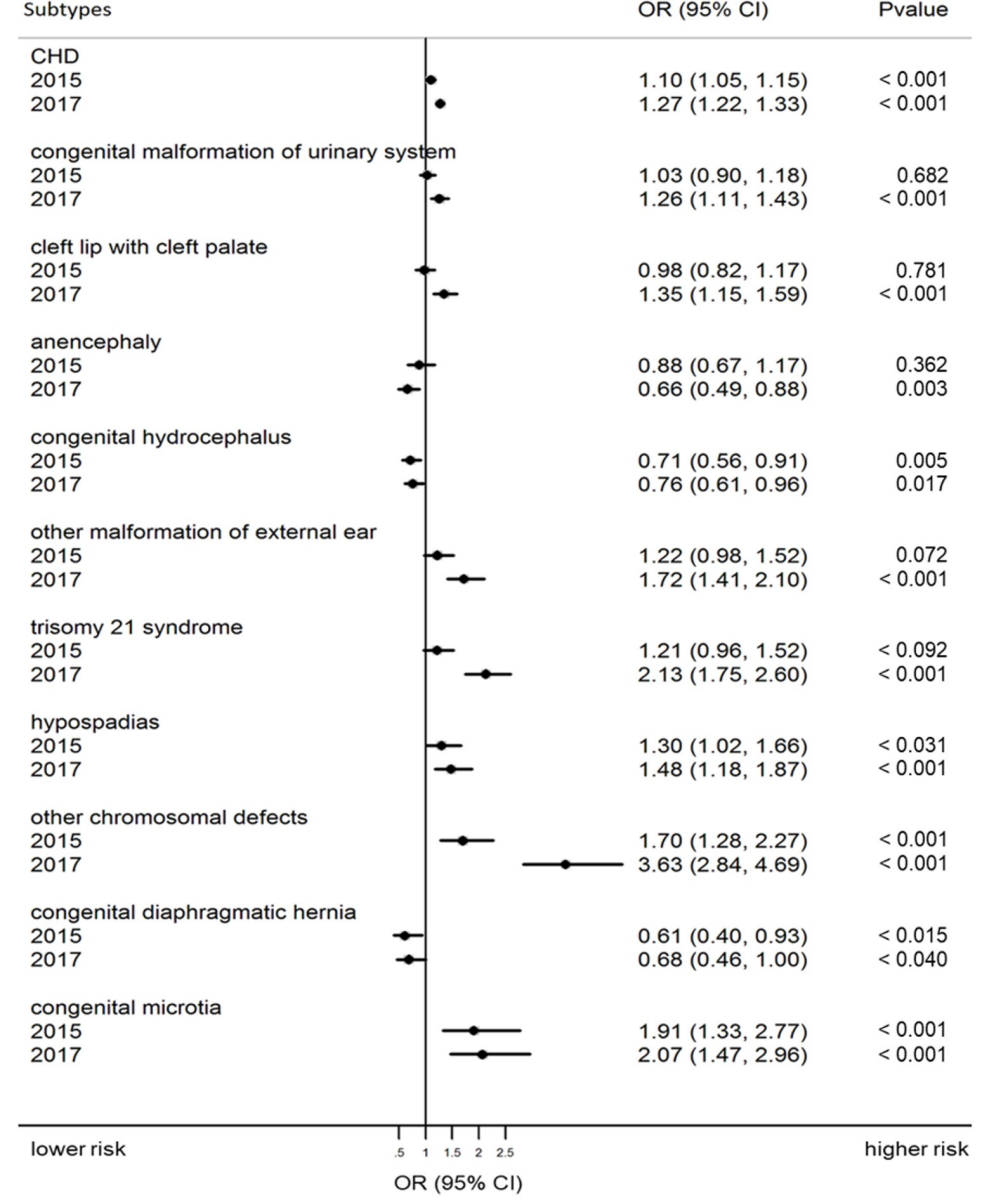
Crude ORs (95% CI) and *P* values for the association between birth policy changes and prevalence of BD subtypes with significant changes in total births (in 2013, 2015, and 2017). ORs and 95% CIs were calculated from exposures (policy changes) and cases (BDs), with 2013 being defined as the reference group. *P* values were derived from chi-squared tests. Abbreviations: CHD, congenital heart defect; CI, confidence interval; OR, odds ratio.

Changes in the risks of BD subtypes across policy changes are presented as ORs (95% CI) with *P* values in S1 Table. Subtypes with significant changes are shown in Fig 2. In the partial and universal two-child policy period, the risk of CHD (partial: OR = 1.10, 95% CI 1.05–1.15, *P* < 0.001; universal: OR = 1.27, 95% CI 1.22–1.33, *P* < 0.001), hypospadias (partial: OR = 1.30, 95% CI 1.02–1.66, *P* = 0.031; universal: OR = 1.48, 95% CI 1.18–1.87, *P* < 0.001), other chromosomal defects (partial: OR = 1.70, 95% CI 1.28–2.27, *P* < 0.001; universal: OR = 3.63, 95% CI 2.84–4.69, *P* < 0.001), and congenital microtia (partial: OR = 1.91, 95% CI 1.33–2.77, *P* < 0.001; universal: OR = 2.07, 95% CI 1.47–2.96, *P* < 0.001) increased significantly compared with risks in the one-child policy period. During the universal two-child policy period, the risk of congenital malformation of the urinary system (OR = 1.26, 95% CI 1.11–1.43, *P* < 0.001), cleft lip with cleft palate (OR = 1.35, 95% CI 1.15–1.59, *P* < 0.001), other malformation of the external ear (OR = 1.72, 95% CI 1.41–2.10, *P* < 0.001), and trisomy 21 syndrome (OR = 2.13, 95% CI 1.75–2.60, *P* < 0.001) also increased significantly compared with risk during the one-child policy period. In the partial and universal two-child policy periods, the risk of congenital hydrocephalus (partial: OR = 0.71, 95% CI 0.56–0.91, *P* = 0.005; universal: OR = 0.76, 95% CI 0.61–0.96, *P* = 0.017) and congenital diaphragmatic hernia (partial: OR = 0.61, 95% CI 0.40–0.93, *P* = 0.015; universal: OR = 0.68, 95% CI 0.46–1.00, *P* = 0.040) decreased significantly compared with risk in the one-child policy period. The risk of anencephaly also decreased significantly during the partial two-child policy period in comparison with risk in the one-child policy period (OR = 0.66, 95% CI 0.49–0.88, *P* = 0.003). Table 3 shows the prevalence and ranking of the 25 main types of BD in total births over the period of policy changes. The top four types of BD were the same in all periods: CHD, polydactyly, congenital malformation of the urinary system, and cleft lip with cleft palate. CHDs remained the leading BD across all study periods, with the prevalence increasing from 143.00 per 10,000 births in the one-child policy period to 181.38 per 10,000 births in the universal two-child policy period. CHDs made up 55.12% of the total BDs in the one-child policy period, 56.34% in the partial two-child policy period, and 55.61% in the universal two-child policy period. Trisomy 21 and other chromosomal defects (highlighted in yellow in Table 3) moved up in ranking over the period of policy changes, from 10th and 16th, respectively, in 2013 to fifth and sixth, respectively, in 2017. The ranking of neural tube defects (NTDs) declined, including anencephaly, spina bifida, and encephalocele (highlighted in light purple in Table 3).

**Table 3:** Ranking of 25 BD subtypes in total BDs over the period of policy changes (in 2013, 2015 and 2017).

Ranking	One-child policy period (2013)			Partial two-child policy period (2015)			Universal two-child policy period (2017)		
	BD subtypes	*N*	Prevalence (per 10,000 births)	BD subtypes	*N*	Prevalence (per 10,000 births)	BD subtypes	*N*	Prevalence (per 10,000 births)
1	CHD	3,820	143.00	CHD	4,173	157.31	CHD	5,661	181.38
2	Polydactyly	508	19.02	Polydactyly	532	20.05	Polydactyly	631	20.22
3	Congenital malformation of urinary system	420	15.72	Congenital malformation of urinary system	429	16.17	Congenital malformation of urinary system	619	19.83
4	Cleft lip with cleft palate	256	9.58	Cleft lip with cleft palate	248	9.35	Cleft lip with cleft palate	405	12.98
5	NTDs	217	8.12	NTDs	189	7.12	Trisomy 21 syndrome	360	11.53
	Anencephaly	113	4.23	Anencephaly	99	3.73			
	Spina bifida	71	2.66	Spina bifida	54	2.04			
	Encephalocele	33	1.24	Encephalocele	36	1.36			
6	Syndactyly	171	6.40	Other malformation of external ear	186	7.01	Other chromosomal defects	339	10.86
7	Congenital hydrocephalus	165	6.18	Trisomy 21 syndrome	174	6.56	Other malformation of external ear	309	9.90
8	Congenital talipes equinovarus	155	5.80	Syndactyly	166	6.26	Congenital talipes equinovarus	221	7.08
9	Other malformation of external ear	154	5.77	Congenital talipes equinovarus	164	6.18	NTDs	211	6.76
							Anencephaly	87	2.79
							Spina bifida	72	2.31
							Encephalocele	52	1.67
10	Trisomy 21 syndrome	145	5.43	Hypospadias	157	5.92	Hypospadias	211	6.76
11	Cleft lip without cleft palate	142	5.32	Cleft lip without cleft palate	150	5.65	Syndactyly	202	6.47
12	Hypospadias	122	4.57	Other chromosomal defects	135	5.09	Congenital hydrocephalus	147	4.71
13	Cleft palate without cleft lip	107	4.01	Congenital hydrocephalus	117	4.41	Cleft lip without cleft palate	140	4.49
14	Limb reduction defects	106	3.97	Cleft palate without cleft lip	112	4.22	Congenital atresia of rectum and anus	119	3.81
15	Congenital atresia of rectum and anus	82	3.07	Limb reduction defects	105	3.96	Cleft palate without cleft lip	117	3.75
16	Other chromosomal defects	80	2.99	Congenital microtia	91	3.43	Congenital microtia	116	3.72
17	Omphalocele	67	2.51	Congenital atresia of rectum and anus	83	3.13	Limb reduction defects	108	3.46
18	Congenital diaphragmatic hernia	64	2.40	Omphalocele	79	2.98	Omphalocele	86	2.76
19	Gastroschisis	54	2.02	Gastroschisis	47	1.77	Gastroschisis	63	2.02
20	Congenital microtia	48	1.80	Congenital diaphragmatic hernia	39	1.47	Congenital diaphragmatic hernia	51	1.63
21	Congenital esophageal atresia	34	1.27	Congenital esophageal atresia	22	0.83	Congenital esophageal atresia	39	1.25
22	Conjoined twins	13	0.49	Conjoined twins	6	0.23	Conjoined twins	22	0.70
23	Exstrophy of urinary bladder	0	0.00	Exstrophy of urinary bladder	3	0.11	Exstrophy of urinary bladder	2	0.06

Note: yellow indicates chromosomal defects, and light purple indicates NTDs.

Abbreviations: BD, birth defect; CHD, congenital heart defect; NTD, neural tube defect

In further analyses by gestational age, chromosomal defects consistently ranked high in infants with BDs born before 28 gestational weeks (S2 Table). Correspondingly, these were ranked relatively low among BDs in infants born at 28 gestational weeks or later (S3 Table).

### Changes in the characteristics of total births with BDs

Table 4 presents the characteristics of total births with BDs. Significant differences in the distributions of maternal age, gravidity, parity, maternal region, birth weight, number of embryos, BD prognosis, and time of diagnosis were found over the years of policy changes (all *P* < 0.05). Among total BDs, the proportion of births with BDs to women aged 30–34 and ≥35 increased by 18.32% and 73.22%, respectively, whereas the proportion of births with BDs to women aged 20–24 and 25–29 decreased from 2013 to 2017. The prenatal diagnosis rate increased by 3.6 percentage points (95% CI 2.2–5.1, *P* < 0.001), from 31.10% in 2013 to 34.72% in 2017. The average time of prenatal diagnosis was 1.88 weeks earlier (95% CI 1.81–1.95, *P* < 0.001) from 2013 to 2017.

**Table 4:** Changes in characteristics of total infants with BDs (in 2013, 2015, and 2017).

Variables	One-child policy period (2013)		Partial two-child policy period (2015)		Universal two-child policy period (2017)		Cramér’s V/*η*2	*P* Value
	*N*/mean	Percent	*N*/mean	Percent	*N*/mean	Percent		
**Total BDs**	6,570	100.00	7,026	100.00	9,499	100.00		-
**Age**							**0.093**	**<0.001**
<20	210	3.20	228	3.25	158	1.66		
20–24	1,416	21.55	1,291	18.37	1,390	14.63		
25–29	2,729	41.54	2,900	41.28	3,567	37.55		
30–34	1,467	22.33	1,618	23.03	2,510	26.42		
≥35	748	11.39	989	14.08	1,874	19.73		
**Gravidity (5 missing)**							**0.082**	**<0.001**
1	2,597	39.53	2,504	35.64	2,868	30.19		
≥2	3,973	60.47	4,522	64.36	6,626	69.75		
**Parity (5 missing)**							**0.011**	**<0.001**
Nulliparous	635	9.67	716	10.19	897	9.44		
Multiparous	5,935	90.33	6,310	89.81	8,597	90.55		
**Maternal region (2 missing)**							**0.207**	**<0.001**
Urban	3,102	47.21	4,108	58.47	6,803	71.62		
Rural	3,468	52.79	2,918	41.53	2,694	28.36		
**Infant sex**							0.012	0.068
Male	3,540	53.88	3,869	55.07	5,224	55.00		
Female	2,815	42.85	2,935	41.77	3,928	41.35		
Unknown	213	3.24	222	3.16	347	3.65		
No information	2	0.03	0	0.00	0	0.00		
**Low birth weight (75 missing)**	2,586	39.52	2,923	41.60	4,056	42.92	**0.028**	**<0.001**
**Number of embryos**							**0.034**	**<0.001**
Single birth	6,203	94.41	6,520	92.80	8,989	94.63		
Multiple births	367	5.59	506	7.20	510	5.37		
**Prognosis (26 missing)**							**0.021**	**<0.001**
Live birth	4,754	72.39	5,260	74.86	6,893	72.74		
Early fetus loss and stillbirths	1,718	26.16	1,672	23.80	2,486	26.23		
Early neonatal deaths	95	1.45	94	1.34	97	1.02	**0.017**	**0.013 (trend)**
**Time of diagnosis (4 missing)**							**0.053**	**<0.001**
Postpartum	4,527	68.90	4,995	71.09	6,197	65.24		
Prenatal	2,043	31.10	2,031	28.91	3,298	34.72		
Week (225 missing)	25.97*	6.36	25.13^#^	5.93	24.26^$^	5.81	**0.011**	**<0.001**

Cramér’s V and corresponding *P* values were derived from chi-squared tests. *η2* and corresponding *P* values were derived from ANOVA. *P*_trend_ was derived from trend analyses.

*Significant differences between one-child policy period and universal two-child policy period, *P* < 0.05.

^#^Significant differences between one-child policy period and partial two-child policy period, *P* < 0.05.

^$^Significant differences between partial two-child policy period and universal two-child policy period, *P* < 0.05.

Abbreviations: ANOVA, analysis of variance; BD, birth defect

### Changes in BDs among infants born before 28 gestational weeks

Table 5 presents characteristics of infants with BDs who were born before 28 gestational weeks. There were significant differences over the time frame of policy changes in birth weight and the distributions of maternal age, gravidity, parity, maternal region, infant sex, number of births, BD prognosis, and time of diagnosis (all *P* < 0.05). Compared with the one-child policy period, birth weight was 45.13 grams (95% CI 14.28–75.99, *P* = 0.004) lower in the partial two-child policy and 65.95 grams (95% CI 38.81–93.08, *P* < 0.001) lower in the universal two-child policy periods. The proportion of births to women aged ≥35 years approximately doubled, from 12.66% during the one-child policy period to 25.02% in the universal two-child policy period. The change was much greater than that in total BDs (from 11.39% to 19.73%, Table 4). More than 96.39% of infants with BDs who were born before 28 gestational weeks were diagnosed prenatally. The average time of prenatal diagnosis was significantly earlier in the universal two-child policy period than in the partial two-child policy period (*P* < 0.05). The live birth rate for BDs in infants born before 28 gestational weeks increased sharply, from 1.29% during the one-child policy period to 11.45% during the universal two-child policy period. The proportion of early fetal loss decreased during this time.

**Table 5:** Changes in characteristics of infants with BDs born before 28 gestational weeks (in 2013, 2015 and 2017).

Variables	One-child policy period (2013)		Partial two-child policy period (2015)		Universal two-child policy period (2017)		Cramér’s V /*η*2	*P* value
	*N*/mean	Percent/SD	*N*/mean	Percent/SD	*N*/mean	Percent/SD		
**BDs born before 28 weeks**	1,090	100.00	1,248	100.00	2,214	100.00	**-**	**-**
**Maternal age**							**0.112**	**<0.001**
<20	21	1.93	37	2.96	27	1.22		
20–24	232	21.28	220	17.63	331	14.95		
25–29	445	40.83	505	40.46	758	34.24		
30–34	254	23.30	292	23.40	544	24.57		
≥35	138	12.66	194	15.54	554	25.02		
**Gravidity**							**0.120**	**<0.001**
1	425	38.99	429	34.38	574	25.93		
≥2	665	61.01	819	65.63	1,640	74.07		
**Parity**							**0.152**	**<0.001**
Nulliparous	556	51.01	610	48.88	774	34.96		
Multiparous	534	49.00	638	51.12	1,440	65.05		
**Maternal region**							**0.178**	**<0.001**
Urban	515	47.25	729	58.41	1,521	68.70		
Rural	575	52.75	519	41.59	693	31.30		
**Infant sex**							**0.054**	**<0.001**
Male	460	42.20	610	48.88	1,141	51.54		
Female	436	40.00	430	34.46	742	33.51		
Unknown	194	17.80	208	16.67	331	14.95		
**Birth weight (68 missing)**	737.29*	387.94	692.15^#^	368.10	671.34	360.97	**0.005**	**0.023**
**Number of embryos**							**0.018**	**<0.001**
Single birth	1,055	96.79	1,217	97.52	2,158	97.47		
Multiple births	35	3.21	31	2.48	56	2.53		
**Prognosis (15 missing)**							**0.197**	**<0.001**
Live birth	14	1.29	28	2.24	252	11.45		
Early fetus loss	1,071	98.35	1,208	96.79	1,942	88.27		
**Time of diagnosis (1 missing)**							**0.063**	**<0.001**
Postpartum	24	2.20	45	3.61	31	1.40		
Prenatal	1,066	97.80	1,203	96.39	2,182	98.60		
Week (152 missing)	21.66	3.90	21.84	3.49	21.53^$^	3.86	**0.001**	**<0.001**

Cramér’s V and corresponding *P* values were derived from chi-squared tests. η2 and corresponding *P* values were derived from ANOVA.

*Significant differences between one-child policy period and universal two-child policy period, *P* < 0.05.

^#^Significant differences between one-child policy period and partial two-child policy period, *P* < 0.05.

^$^Significant differences between partial two-child policy period and universal two-child policy period, *P* < 0.05.

Abbreviation: ANOVA, analysis of variance; BD, birth defect; SD, standard deviation

## Discussion

Using data from over 1.26 million births in 90 representative hospitals of Zhejiang Province, we found that the proportion of women with advanced maternal age increased by 85.68% with policy changes, from 8.52% in 2013 to 15.82% in 2017, whereas it remained stable prior to the policy changes. The proportion of mothers living in urban areas also increased. For changes in BDs, we found significant increases in the prevalence of total and age-related BDs over the years of policy changes. According to the analysis by maternal age, risks of total BDs increased in women aged 20–24, 25–29, 30–34, and ≥35 over the years of policy changes. In terms of BD subtypes, CHD remained the predominant abnormality in the period of changing birth policy, with an increasing trend in prevalence. For birth outcomes, infants with BDs born before 28 gestational weeks demonstrated a sharp increase in the live birth rate throughout the period of policy changes.

Among our findings, of particular interest was the sharp increase in the proportion of women with advanced maternal age across the birth policy changes (8.52% in the one-child policy period to 15.82% in the universal two-child policy period). Although postponing childbirth has increased globally [26,27], the new birth policy in China may have encouraged fertility desires and behaviors among older women. To better identify the effects of these birth policy changes, we reviewed data for the proportion of childbearing women aged ≥35 years before initiation of the new policies. This proportion remained stable from 2009 to 2013, prior to policy changes. However, births to women over age 35 years increased sharply from 2013 to 2017, once the two-child policies were introduced. This finding was also evidenced in Chinese national surveillance data showing that births to women older than 35 years increased from 7.8% to 10.9% since relaxation of the one-child policy [11].

In 2017 (the universal two-child policy period), BDs affected 304.36 per 10,000 births in Zhejiang Province. This prevalence is comparable with reports from the US National Birth Defects Prevention Network (NBDPN), European Surveillance of Congenital Anomalies (EUROCAT), and the national prevalence of Thailand (200–300 per 10,000 births); this prevalence is lower than the national level of Korea (446.3 per 10,000 births) and arctic regions of Russia (361 per 10,000 births) [17,18,28–32]. The variations among the different studies may be attributable to differences in sociodemographic characteristics, inclusion criteria, diagnostic technologies, and methods of case ascertainment. Most countries or regions included in the NBDPN and EUROCAT registries have active population-based programs with multiple data resources and long-term follow-up [17,18]. Although the BD registry in Thailand is hospital based, it includes live births within 1 year after birth [32]. Our study used a hospital-based BD tracking system registering live births, stillbirths, and pregnancy terminations for fetal anomaly at any gestational age.

In terms of BD subtypes among all births, the four most frequent subtypes did not change with changes in the birth policy. CHDs were the most common defect, which is consistent with many previous studies [29,32,33]. The prevalence of CHDs was 181.38 per 10,000 births in 2017, which is far higher than those in most other areas. Of every 10,000 births, 100 births in the US, 65.9 in India, and 137.1 in Norway have CHDs [28,33–36]. Most regions of Zhejiang Province have easy access to echocardiography for routine pediatric examination of newborns. Some diagnoses of CHDs may be revised with increased age of the child. For example, EUROCAT recommends reporting an atrial septum defect 6 months postpartum [18]. Thus, there might be some heterogeneity in CHDs across different studies and an overestimation of CHDs in our study, and the development of diagnostic technology might contribute to the increase in prevalence of CHD.

An increase in congenital anomalies, such as trisomy 21 and other chromosomal anomalies, was found in our study. This finding was consistent with our previous study [37]. Increased maternal age could be one of the explanations. In the present study, the prevalence of trisomy 21 increased from 5.43 in 2013 to 11.53 per 10,000 births in 2017. Globally, the prevalence of trisomy 21 varies among regions; for example, 10.03 per 10,000 births in Europe, 14 per 10,000 births in Austria, 14.5 per 10,000 births in the US, and 1.99 per 10,000 perinatal births in China [17,18,38–40]. However, the proportion of births to women aged ≥35 years in our study in 2017 (15.82%) was similar to that in Austria during 1980–2013 (14%) and slightly lower than Europe (19%) but was much higher than national data for China collected in 2011 (9.20%) [39,40].

As a well-known risk factor [41], increased maternal age might explain part—but not all—of the increases in BDs in the total population and in the older maternal age group and the increase in chromosomal defects. However, there were other explanations, given that the risks for BDs remained increased not only in the older maternal age groups but also in the young maternal age groups following policy changes. Improvements in prenatal screening and prenatal diagnostic technology, as well as changes in the socioeconomic status of parents, could be important factors explaining the increase in total BDs and BD subtypes across all maternal age groups. Coinciding with the start of the two-child policy in 2016, the Chinese government approved noninvasive prenatal testing at the national level. In Zhejiang Province, access to healthcare, maternal serum, and ultrasound screening in the first and second trimesters, as well as clinical assessment prenatally and at birth, have become routinely available and have been improved [42]. Moreover, awareness of prenatal healthcare among women has grown. These factors may also contribute to the increase in total BDs and BD subtypes and the prenatal diagnosis rate of BDs in this study. Our observation that a higher proportion of mothers live in urban areas with birth policy changes (Table 1) might contribute to the increases in BDs because of the more developed diagnostic technology and higher accessibility to healthcare facilities in cities. Previous studies have also reported that lower income, lower educational level, and certain occupations of parents, such as working in cleaning or healthcare occupations, are associated with higher risks for BD subtypes [15,43]. Further studies with detailed information on socioeconomic status are thus needed to determine whether socioeconomic status plays a role in the association between birth policy changes and BDs. Other factors, e.g., increasing multiple births, genetic changes, and environmental exposures, might also contribute to the results [28–30,44]. The impact of improved technology on the surveillance system over time could also have contributed to the increase in total BDs and BD subtypes. For example, the development of diagnostic technologies may have improved the detection rate for minor malformations and chromosomal defects. The development of information technology may also have improved the quality of surveillance.

Conversely, the findings of our study indicate that certain BD subtypes decreased. Enhanced public awareness regarding the prevention of severe BDs from the parents and the measures that have been developed and adopted by the government could be two possible explanations for this finding. Folic acid intake by women before conception and during the first trimester of their pregnancies has been found to be efficacious in preventing NTDs in their offspring [45]. The Zhejiang government provided free folic acid and free consultation service on BD prevention in premarital tests. The finding of a decrease in NTDs was consistent with those of studies worldwide [46,47] and could be attributed to the promotion of folic acid intervention and to greater compliance with the guidelines among parents. Furthermore, in response to possible challenges relating to the birth policy changes, the Chinese government has implemented free prepregnancy screening in rural areas at a national scale and funded a disease-screening program for newborns in poor areas that commenced in 2013 [48,49]. In addition, the improvement in the health literacy of individuals has encouraged greater utilization of healthcare facilities. Other factors associated with these BD subtypes may also have contributed to their decrease. For example, previous studies have reported that genetic and lifestyle factors and socioeconomic status are associated with NTD [50,51]. Further studies are needed to investigate whether these factors play roles in the decrease in certain BD subtypes with birth policy changes.

With the progress in prenatal diagnosis and treatment for infants with BDs, a consistently high live birth rate for total BDs was found in this study (Table 4). In particular, the live birth rate among infants with BDs born before 28 gestational weeks increased from 1.29% during the one-child policy period to 11.45% during the universal two-child policy period. In Europe and the US, the reported BD prevalence includes births irrespective of gestational age [17,18]. However, most studies or monitoring reports of BDs or stillbirths in China have mainly focused on births at 28 completed weeks or later [24,28,34] because the perinatal period starts at 28 complete weeks in China [25]. Nevertheless, WHO suggests that in some developed countries, the perinatal period commences at 22 completed weeks’ gestation [52]. A study in Beijing showed that NTDs at 28 weeks represented only 29.1% of those diagnosed at 13 weeks, highlighting the importance of BD detection prior to 28 completed weeks’ gestation [53]. However, there are few data on BDs in infants born before 28 weeks’ gestation in China. In our study, among those infants from this group who lived, the proportion of CHDs, chromosomal anomalies, and body surface anomalies increased over the time frame of the policy changes. The current Chinese literature on BDs predominantly focuses on perinatal, live births in China [28,34,54,55]. Studies on infants born before 28 gestational weeks whose BDs were identified in the pre-prenatal period are scarce. To better estimate the burden of disease due to BDs, pre-prenatal anomalies, particularly those among infants born at an early gestational age, merit additional attention.

This study has two main strengths. First, the large sample size led to stable and reliable study results. The total number of more than 1.26 million births allowed us to accurately identify several rare BDs. Second, the analysis focused on BDs in three independent periods in line with the birth policy changes. This provides a comprehensive estimation of the associations of policy changes with BDs, yielding evidence for governmental support and resource allocation. There were several limitations of our study as well. First, this observational study could not prove a causal association between the increasing BD prevalence and policy changes. Second, the data used in this study were from a hospital-based BD registry system in Zhejiang. Although a multistage sampling method was used to select hospitals, and the hospital delivery rate in Zhejiang Province was already close to 100% in 2015 [56], a potential lack of representativeness exists. The generalizability of the findings to some other areas of China might be limited owing to the large variation among provinces in socioeconomic development and cultural characteristics. Third, we were unable to identify the infants with BDs born to women who decided to become pregnant based on policy changes because we lacked information on parity of births without BDs. Finally, BDs are multifactorial disorders [16]. However, we could not perform a multivariate analysis for other factors associated with BDs because no individual information for births without BDs was collected—for example, socioeconomic factors, individual genetic backgrounds, and environmental determinants. Further studies are therefore needed to determine whether these factors play a role in the associations between birth policy changes and BDs.

We observed significant changes in maternal characteristics, such as a near double increase in advanced maternal age, and the changes of BDs after the implementation of China’s new two-child policy. Despite the increase in overall prevalence of BDs, CHDs, and chromosomal anomalies, there has been a reduction in lethal anomalies. The increases in risks for BDs in younger maternal age groups also suggested that some other factors (in addition to advanced maternal age) might contribute to the increases in BDs. The prenatal diagnosis rate for BDs has improved over the time frame of the policy changes. The sharp increase in the live birth rate among infants with BDs born before 28 gestational weeks suggests that healthcare for very preterm infants with BDs, as well as updating the definition of the perinatal period, may be warranted in the future. In addition, advocating for improved healthcare for infants with BDs is urgent. Further studies on long-term associations between these birth policy changes and BDs are needed in the future.

## Supporting information

S1 Checklist STROBE checklist for cohort, case-control, and cross-sectional studies (combined). STROBE, strengthening the reporting of observational studies in epidemiology.
(DOCX)

S1 Table Crude ORs (95% CIs) and *P* values for the associations between birth policy changes and BD subtypes in total births (in 2013, 2015, and 2017). BD, birth defect; CI, confidence interval; OR, odds ratio.
(DOCX)

S2 Table Ranking of 25 BD subtypes among infants with BDs born before 28 gestational weeks (in 2013, 2015, and 2017). BD, birth defect.
(DOCX)

S3 Table Ranking of 25 BD subtypes among infants with BDs born at ≥28 gestational weeks (in 2013, 2015, and 2017). BD, birth defect.
(DOCX)

## Abbreviations

ANOVA: analysis of variance
BD: birth defect
CHD: congenital heart defect
CI: confidence interval
EUROCAT: European Surveillance of Congenital Anomalies
GRP: gross regional product
NBDPN: National Birth Defects Prevention Network
NTD: neural tube defect
OR: odds ratio
SD: standard deviation
