# Supplementary material for: Changes in maternal age and prevalence of congenital anomalies during the enactment of China's universal two-child policy (2013–2017) in Zhejiang Province, China: An observational study
Source: PLoS Med. 2020 Feb 24;17(2):e1003047. doi: 10.1371/journal.pmed.1003047 (PMC7039412; doi:10.1371/journal.pmed.1003047)
Supplement: S1 Checklist — STROBE, strengthening the reporting of observational studies in epidemiology. (DOCX) [file pmed.1003047.s001.docx]

STROBE Statement—checklist of items that should be included in reports of observational studies

|  | Item No | Recommendation | Paragraph No |
| --- | --- | --- | --- |
| **Title and abstract** | 1 | (*a*) Indicate the study’s design with a commonly used term in the title or the abstract | Title. |
|  |  | (*b*) Provide in the abstract an informative and balanced summary of what was done and what was found | Abstract. |
| Introduction | | |  |
| Background/rationale | 2 | Explain the scientific background and rationale for the investigation being reported | Introduction section: paragraph 1-2. |
| Objectives | 3 | State specific objectives, including any prespecified hypotheses | Methods section: paragraph 1 “Study design and population”. |
| Methods | | |  |
| Study design | 4 | Present key elements of study design early in the paper | Method section: paragraph 1 “Study design and population”. |
| Setting | 5 | Describe the setting, locations, and relevant dates, including periods of recruitment, exposure, follow-up, and data collection | Method section: paragraph 1-2 “Study design and population” and “Data collection”. |
| Participants | 6 | (*a*) Give the eligibility criteria, and the sources and methods of selection of participants | Method section: paragraph 1 “Study design and population”. |
|  |  | (*b*) *Cohort study*—For matched studies, give matching criteria and number of exposed and unexposed  *Case-control study*—For matched studies, give matching criteria and the number of controls per case | NA |
| Variables | 7 | Clearly define all outcomes, exposures, predictors, potential confounders, and effect modifiers. Give diagnostic criteria, if applicable | Method section: paragraph 3-4 “Criteria of BDs diagnosis and variables definition”. |
| Data sources/ measurement | 8* | For each variable of interest, give sources of data and details of methods of assessment (measurement). Describe comparability of assessment methods if there is more than one group | Method section: paragraph 2-4 “Data collection” and “Criteria of BDs diagnosis and variables definition”. |
| Bias | 9 | Describe any efforts to address potential sources of bias | Method section: paragraph 2 “Data collection” . |
| Study size | 10 | Explain how the study size was arrived at | Method section: paragraph 1 “Study design and population”. |
| Quantitative variables | 11 | Explain how quantitative variables were handled in the analyses. If applicable, describe which groupings were chosen and why | Method section: paragraph 3 “Criteria of BDs diagnosis and variables definition”. |
| Statistical methods | 12 | (*a*) Describe all statistical methods, including those used to control for confounding | Method section: paragraph 5 “Statistical analysis”. |
|  |  | (*b*) Describe any methods used to examine subgroups and interactions | Method section: paragraph 5 “Statistical analysis”. |
|  |  | (*c*) Explain how missing data were addressed | Results section: Table1. |
|  |  | (*d*) If applicable, describe analytical methods taking account of sampling strategy |  |
|  |  | (*e*) Describe any sensitivity analyses |  |
| Results | | |  |
| Participants | 13* | (a) Report numbers of individuals at each stage of study—eg numbers potentially eligible, examined for eligibility, confirmed eligible, included in the study, completing follow-up, and analysed | Results section: paragraph 1, Table 1. |
|  |  | (b) Give reasons for non-participation at each stage | Results section: Table 1. |
|  |  | (c) Consider use of a flow diagram |  |
| Descriptive data | 14* | (a) Give characteristics of study participants (eg demographic, clinical, social) and information on exposures and potential confounders | Results section: paragraph 1, Table 1, S4 Table. |
|  |  | (b) Indicate number of participants with missing data for each variable of interest | Results section: Table 1. |
| Outcome data | 15* | Report numbers of outcome events or summary measures | Results section: Table 1, Figure 1, Table 3. |
| Main results | 16 | (*a*) Give unadjusted estimates and, if applicable, confounder-adjusted estimates and their precision (eg, 95% confidence interval). Make clear which confounders were adjusted for and why they were included | Results section: Table 2. |
|  |  | (*b*) Report category boundaries when continuous variables were categorized | Method section: paragraph 3-4 “Criteria of BDs diagnosis and variables definition”. |
|  |  | (*c*) If relevant, consider translating estimates of relative risk into absolute risk for a meaningful time period |  |
| Other analyses | 17 | Report other analyses done—eg analyses of subgroups and interactions, and sensitivity analyses | Results section: Fig 2, S1 Table, S2 Table, S3 Table, Table 5. |
| Discussion | | |  |
| Key results | 18 | Summarise key results with reference to study objectives | Discussion section: paragraph 1. |
| Limitations | 19 | Discuss limitations of the study, taking into account sources of potential bias or imprecision. Discuss both direction and magnitude of any potential bias | Discussion section: “Strengths and limitations”. |
| Interpretation | 20 | Give a cautious overall interpretation of results considering objectives, limitations, multiplicity of analyses, results from similar studies, and other relevant evidence | Discussion section: “Conclusion”. |
| Generalisability | 21 | Discuss the generalisability (external validity) of the study results | Discussion section: “Findings of this study”. |
| Other information | | |  |
| Funding | 22 | Give the source of funding and the role of the funders for the present study and, if applicable, for the original study on which the present article is based | Financial Disclosure section. |

*Give information separately for cases and controls in case-control studies and, if applicable, for exposed and unexposed groups in cohort and cross-sectional studies.

**Note:** An Explanation and Elaboration article discusses each checklist item and gives methodological background and published examples of transparent reporting. The STROBE checklist is best used in conjunction with this article (freely available on the Web sites of PLoS Medicine at http://www.plosmedicine.org/, Annals of Internal Medicine at http://www.annals.org/, and Epidemiology at http://www.epidem.com/). Information on the STROBE Initiative is available at www.strobe-statement.org.
