# Supplementary material for: Changes in maternal age and prevalence of congenital anomalies during the enactment of China's universal two-child policy (2013–2017) in Zhejiang Province, China: An observational study
Source: PLoS Med. 2020 Feb 24;17(2):e1003047. doi: 10.1371/journal.pmed.1003047 (PMC7039412; doi:10.1371/journal.pmed.1003047)
Supplement: S1 Table — BD, birth defect; CI, confidence interval; OR, odds ratio. (DOCX) [file pmed.1003047.s002.docx]

S1 Table. Crude ORs (95% CIs) and *P* values for the associations between birth policy changes and BD subtypes in total births (in 2013, 2015, and 2017).

| **Subtype of BD** | **One-child policy period (2013)** | |  | **Partial two-child policy period (2015)** | |  | **Universal two-child policy period (2017)** | |
| --- | --- | --- | --- | --- | --- | --- | --- | --- |
|  | **OR(95%CI)** | ***P*** |  | **OR(95%CI)** | ***P*** |  | **OR(95%CI)** | ***P*** |
| CHD | 1 | - |  | **1.10(1.05 to 1.15)** | **<0.0001** |  | **1.27(1.22 to 1.33)** | **<0.0001** |
| polydactyly | 1 | - |  | 1.05(0.93 to 1.19) | 0.3914 |  | 1.06(0.94 to 1.20) | 0.3039 |
| congenital malformation of urinary system | 1 | - |  | 1.03(0.90 to 1.18) | 0.6815 |  | **1.26(1.11 to 1.43)** | **0.0002** |
| cleft lip with cleft palate | 1 | - |  | 0.98(0.82 to 1.17) | 0.7806 |  | **1.35(1.15 to 1.59)** | **0.0001** |
| NTDs | 1 | - |  | 0.88(0.72 to 1.07) | 0.1868 |  | 0.83(0.69 to 1.01) | 0.0571 |
| anencephaly | 1 | - |  | 0.88(0.67 to 1.17) | 0.3622 |  | **0.66(0.49 to 0.88)** | **0.0032** |
| spina bifida | 1 | - |  | 0.77(0.53 to 1.11) | 0.1384 |  | 0.87(0.62 to 1.22) | 0.3967 |
| encephalocele | 1 | - |  | 1.1(0.67 to 1.82) | 0.6965 |  | 1.35(0.86 to 2.15) | 0.1773 |
| syndactyly | 1 | - |  | 0.98(0.78 to 1.22) | 0.8347 |  | 1.01(0.82 to 1.25) | 0.9157 |
| congenital hydrocephalus | 1 | - |  | **0.71(0.56 to 0.91)** | **0.0051** |  | **0.76(0.61 to 0.96)** | **0.0165** |
| congenital talipes equinovarus | 1 | - |  | 1.07(0.85 to 1.34) | 0.5713 |  | 1.22(0.99 to 1.51) | 0.0568 |
| other malformation of external ear | 1 | - |  | 1.22(0.98 to 1.52) | 0.0719 |  | **1.72(1.41 to 2.10)** | **<0.0001** |
| trisomy 21 syndrome | 1 | - |  | 1.21(0.96 to 1.52) | 0.0918 |  | **2.13(1.75 to 2.60)** | **<0.0001** |
| cleft lip without cleft palate | 1 | - |  | 1.06(0.84 to 1.35) | 0.5977 |  | 0.84(0.66 to 1.07) | 0.1534 |
| hypospadias | 1 | - |  | **1.30(1.02 to 1.66)** | **0.0312** |  | **1.48(1.18 to 1.87)** | **0.0005** |
| cleft palate without cleft lip | 1 | - |  | 1.05(0.80 to 1.39) | 0.6970 |  | 0.94(0.71 to 1.23) | 0.6202 |
| limb reduction defects | 1 | - |  | 1.00(0.75 to 1.32) | 0.9853 |  | 0.87(0.66 to 1.15) | 0.3162 |
| congenital atresia of rectum and anus | 1 | - |  | 1.02(0.74 to 1.40) | 0.9025 |  | 1.24(0.93 to 1.67) | 0.1301 |
| other chromosomal defects | 1 | - |  | **1.70(1.28 to 2.27)** | **0.0001** |  | **3.63(2.84 to 4.69)** | **<0.0001** |
| omphalocele | 1 | - |  | 1.19(0.85 to 1.67) | 0.3006 |  | 1.10(0.79 to 1.53) | 0.5636 |
| congenital diaphragmatic hernia | 1 | - |  | **0.61(0.40 to 0.93)** | **0.0152** |  | **0.68(0.46 to 1.00)** | **0.0402** |
| gastroschisis | 1 | - |  | 0.88(0.58 to 1.32) | 0.5082 |  | 1.00(0.68 to 1.46) | 0.9938 |
| congenital microtia | 1 | - |  | **1.91(1.33 to 2.77)** | **0.0002** |  | **2.07(1.47 to 2.96)** | **<0.0001** |
| congenital esophageal atresia | 1 | - |  | 0.65(0.36 to 1.15) | 0.1146 |  | 0.98(0.60 to 1.60) | 0.9375 |
| conjoined twins | 1 | - |  | 0.46(0.14 to 1.31) | 0.1117 |  | 1.45(0.70 to 3.13) | 0.2868 |
| exstrophy of urinary bladder^*^ | - | - |  | - | - |  | - | - |

ORs and 95%CIs were calculated from exposures (policy changes) and cases (BDs). *P* values were derived from chi-square tests.

*Number of births with exstrophy of urinary bladder in 2013 is 0.
