# Supplementary material for: Changes in maternal age and prevalence of congenital anomalies during the enactment of China's universal two-child policy (2013–2017) in Zhejiang Province, China: An observational study
Source: PLoS Med. 2020 Feb 24;17(2):e1003047. doi: 10.1371/journal.pmed.1003047 (PMC7039412; doi:10.1371/journal.pmed.1003047)
Supplement: S2 Table — BD, birth defect. (DOCX) [file pmed.1003047.s003.docx]

**S2 Table. Ranking of 25 BD subtypes among infants with BDs born before 28 gestational weeks (in 2013, 2015, and 2017).**

| **Ranking** | **One-child policy period (2013)** | |  | **Partial two-child policy period (2015)** | |  | **Universal two-child policy period(2017)** | |
| --- | --- | --- | --- | --- | --- | --- | --- | --- |
|  | **BD** **subtypes** | **N** |  | **BD subtypes** | **N** |  | **BD subtypes** | **N** |
| 1 | CHD | 279 |  | CHD | 353 |  | CHD | 800 |
| 2 | cleft lip with cleft palate | 155 |  | cleft lip with cleft palate | 160 |  | cleft lip with cleft palate | 296 |
| 3 | trisomy 21 syndrome | 100 |  | congenital malformation of urinary system | 131 |  | trisomy 21 syndrome | 288 |
| 4 | anencephaly | 97 |  | trisomy 21 syndrome | 115 |  | other chromosomal defect | 232 |
| 5 | congenital malformation of urinary system | 91 |  | other chromosomal defect | 90 |  | congenital malformation of urinary system | 206 |
| 6 | cleft lip without cleft palate | 63 |  | anencephaly | 86 |  | congenital talipes equinovarus | 74 |
| 7 | other chromosomal defect | 59 |  | cleft lip without cleft palate | 82 |  | anencephaly | 72 |
| 8 | congenital talipes equinovarus | 50 |  | omphalocele | 63 |  | omphalocele | 62 |
| 9 | omphalocele | 48 |  | congenital talipes equinovarus | 49 |  | cleft lip without cleft palate | 59 |
| 10 | congenital hydrocephalus | 45 |  | congenital hydrocephalus | 47 |  | limp reduction defects | 55 |
| 11 | limp reduction defects | 43 |  | limp reduction defects | 43 |  | congenital hydrocephalus | 51 |
| 12 | spina bifida | 38 |  | gastroschisis | 39 |  | spina bifida | 51 |
| 13 | gastroschisis | 33 |  | spina bifida | 32 |  | encephalocele | 51 |
| 14 | encephalocele | 25 |  | encephalocele | 29 |  | gastroschisis | 50 |
| 15 | congenital diaphragmatic hernia | 21 |  | polydactyly | 18 |  | congenital diaphragmatic hernia | 25 |
| 16 | polydactyly | 16 |  | congenital diaphragmatic hernia | 14 |  | polydactyly | 20 |
| 17 | conjoined twins | 13 |  | cleft palate without cleft lip | 9 |  | other malformation of external ear | 13 |
| 18 | cleft palate without cleft lip | 8 |  | syndactyly | 6 |  | conjoined twins | 9 |
| 19 | syndactyly | 3 |  | conjoined twins | 5 |  | syndactyly | 8 |
| 20 | congenital atresia of rectum and anus | 2 |  | other malformation of external ear | 4 |  | hypospadias | 8 |
| 21 | congenital esophageal atresia | 2 |  | congenital microtia | 3 |  | congenital atresia of rectum and anus | 6 |
| 22 | congenital microtia | 1 |  | hypospadias | 1 |  | cleft palate without cleft lip | 5 |
| 23 | other malformation of external ear | 0 |  | exstrophy of urinary bladder | 1 |  | congenital microtia | 4 |
| 24 | hypospadias | 0 |  | congenital atresia of rectum and anus | 0 |  | congenital esophageal atresia | 3 |
| 25 | exstrophy of urinary bladder | 0 |  | congenital esophageal atresia | 0 |  | exstrophy of urinary bladder | 1 |

Note: yellow: chromosomal defects; light purple: NTDs.
