# Supplementary material for: Changes in maternal age and prevalence of congenital anomalies during the enactment of China's universal two-child policy (2013–2017) in Zhejiang Province, China: An observational study
Source: PLoS Med. 2020 Feb 24;17(2):e1003047. doi: 10.1371/journal.pmed.1003047 (PMC7039412; doi:10.1371/journal.pmed.1003047)
Supplement: S3 Table — BD, birth defect. (DOCX) [file pmed.1003047.s004.docx]

**S3 Table. Ranking of 25 BD subtypes among infants with BDs born at ≥28 gestational weeks (in 2013, 2015, and 2017).**

| **Ranking** | **One-child policy period (2013)** | |  | **Partial two-child policy period (2015)** | |  | **Universal two-child policy period (2017)** | |
| --- | --- | --- | --- | --- | --- | --- | --- | --- |
|  | **BD** **subtypes** | **N** |  | **BDs subtypes** | **N** |  | **BD subtypes** | **N** |
| 1 | CHD | 3,541 |  | CHD | 3,820 |  | CHD | 4,861 |
| 2 | polydactyly | 492 |  | polydactyly | 514 |  | polydactyly | 611 |
| 3 | congenital malformation of urinary system | 329 |  | congenital malformation of urinary system | 298 |  | congenital malformation of urinary system | 413 |
| 4 | syndactyly | 168 |  | other malformation of external ear | 182 |  | other malformation of external ear | 296 |
| 5 | other malformation of external ear | 154 |  | syndactyly | 160 |  | hypospadias | 203 |
| 6 | hypospadias | 122 |  | hypospadias | 156 |  | syndactyly | 194 |
| 7 | congenital hydrocephalus | 120 |  | congenital talipes equinovarus | 115 |  | congenital talipes equinovarus | 147 |
| 8 | congenital talipes equinovarus | 105 |  | cleft palate without cleft lip | 103 |  | congenital atresia of rectum and anus | 113 |
| 9 | cleft lip with cleft palate | 101 |  | cleft lip with cleft palate | 88 |  | cleft palate without cleft lip | 112 |
| 10 | cleft palate without cleft lip | 99 |  | congenital microtia | 88 |  | congenital microtia | 112 |
| 11 | congenital atresia of rectum and anus | 80 |  | congenital atresia of rectum and anus | 83 |  | cleft lip with cleft palate | 109 |
| 12 | cleft lip without cleft palate | 79 |  | congenital hydrocephalus | 70 |  | other chromosomal defect | 107 |
| 13 | limb reduction defects | 63 |  | cleft lip without cleft palate | 68 |  | congenital hydrocephalus | 96 |
| 14 | congenital microtia | 47 |  | limb reduction defects | 62 |  | cleft lip without cleft palate | 81 |
| 15 | trisomy 21 syndrome | 45 |  | trisomy 21 syndrome | 59 |  | trisomy 21 syndrome | 72 |
| 16 | congenital diaphragmatic hernia | 43 |  | other chromosomal defect | 45 |  | limb reduction defects | 53 |
| 17 | spina bifida | 33 |  | congenital diaphragmatic hernia | 25 |  | congenital esophageal atresia | 36 |
| 18 | congenital esophageal atresia | 32 |  | spina bifida | 22 |  | congenital diaphragmatic hernia | 26 |
| 19 | gastroschisis | 21 |  | congenital esophageal atresia | 22 |  | omphalocele | 24 |
| 20 | other chromosomal defect | 21 |  | omphalocele | 16 |  | spina bifida | 21 |
| 21 | omphalocele | 19 |  | anencephaly | 13 |  | anencephaly | 15 |
| 22 | anencephaly | 16 |  | gastroschisis | 8 |  | gastroschisis | 13 |
| 23 | encephalocele | 8 |  | encephalocele | 7 |  | conjoined twins | 13 |
| 24 | exstrophy of urinary bladder | 0 |  | exstrophy of urinary bladder | 2 |  | encephalocele | 1 |
| 25 | conjoined twins | 0 |  | conjoined twins | 1 |  | exstrophy of urinary bladder | 1 |

Note: yellow: chromosomal defects; light purple: NTDs.
